# An LDV based method to quantify the error of PC-MRI derived Wall Shear Stress measurement

**DOI:** 10.1038/s41598-021-83633-y

**Published:** 2021-02-18

**Authors:** Marco Castagna, Sébastien Levilly, Perrine Paul-Gilloteaux, Saïd Moussaoui, Jean-Marc Rousset, Félicien Bonnefoy, Jérôme Idier, Jean-Michel Serfaty, David Le Touzé

**Affiliations:** 1 LHEEA Lab, École Centrale Nantes, CNRS UMR 6598, 1 rue de la Noë, 44321 Nantes, France; 2 Université de Nantes, CHU Nantes, CNRS UMR 6291, INSERM UMR 1087, L’institut du thorax, 8 quai Moncousu, 44035 Nantes, France; 3 LS2N, École Centrale Nantes, CNRS UMR 6004, 1 rue de la Noë, 44321 Nantes, France; 4 Université de Nantes, CHU Nantes, CNRS UMS 3556, INSERM UMS 016, SFR Santé, 8 quai Moncousu, 44035 Nantes, France

**Keywords:** Biomarkers, Cardiovascular diseases

## Abstract

*Wall Shear Stress* (WSS) has been demonstrated to be a biomarker of the development of atherosclerosis. In vivo assessment of WSS is still challenging, but *4D Flow MRI* represents a promising tool to provide 3D velocity data from which WSS can be calculated. In this study, a system based on *Laser Doppler Velocimetry* (LDV) was developed to validate new improvements of 4D Flow MRI acquisitions and derived WSS computing. A hydraulic circuit was manufactured to allow both 4D Flow MRI and LDV velocity measurements. WSS profiles were calculated with one 2D and one 3D method. Results indicated an excellent agreement between MRI and LDV velocity data, and thus the set-up enabled the evaluation of the improved performances of 3D with respect to the 2D-WSS computation method. To provide a concrete example of the efficacy of this method, the influence of the spatial resolution of MRI data on derived 3D-WSS profiles was investigated. This investigation showed that, with acquisition times compatible with standard clinical conditions, a refined MRI resolution does not improve WSS assessment, if the impact of noise is unreduced. This study represents a reliable basis to validate with LDV WSS calculation methods based on 4D Flow MRI.

## Introduction

Atherosclerosis represents one of the most prevalent cardiovascular diseases. It accounts for 21% of deaths worldwide and implies high social costs^1^. Its risk factors like hypertension, tobacco smoking, diabetes, and hypercholesterolemia are systemic but atherosclerotic plaques occur mainly in specifically placed locations of the arterial system like bifurcation and branching. In these sites, the *Wall Shear Stress* (WSS), the viscous frictional force of blood flowing on arterial walls, can deviate more frequently from physiological ranges^2^. It has been demonstrated that highly sensitive atherosclerotic sites are correlated with low time-average and high spatiotemporal oscillating WSS values, triggering atherosclerotic plaque formation and modulating its progression^3^.

In vivo blood velocity mapping and estimation of related WSS still represent a challenging task. Echocardiography is routinely employed for blood flow diagnosis for its short scan time and patient comfort, but its coverage, accuracy, and resolution are limited by probe acoustical access^4^. In contrast, *Phase-Contrast MRI* (PC MRI) is unaffected by this drawback and since its introduction during the late 1980s, it is gaining more and more prominence. More recently, *4D PC MRI* (or 4D Flow MRI) has been developed, enabling acquisitions of the temporal and spatial evolution of blood velocity patterns with full volumetric coverage. Additionally, anatomical images are recorded to obtain lumen segmentation. Nevertheless, a relatively long time scan and a limited spatiotemporal resolution restrict its clinical applications^4,5^.

Several WSS calculation methods based on PC MRI have been proposed. Assuming a fully developed laminar flow in a circular pipe, WSS can be calculated from the Poiseuille equation. The most robust Poiseuille-based method employed the maximum measured velocity $v_{\mathrm {max}}$, but it did not consider any WSS spatial and temporal features^6^. Several substitute methods based on linear or parabolic fittings of velocity data have been suggested, even if velocity profiles are often not linear or parabolic^7–9^. Stalder et al.^10^ proposed to slice 4D Flow MRI data at determined planes or start from 2D PC MRI data, to contour vascular lumen, and to employ a B-spline interpolation for 3D velocity profiles. Although this method is time effective, it is limited to the selected planes. Potters et al.^11^ proposed instead to consider the whole volume segmentation and to fit velocity close to the boundaries with a smooth piecewise polynomial. Alternatively, Sotelo et al.^12^ calculated WSS with a *Finite Element Method* (FEM) starting from a cubic interpolation of the velocity field on the nodes of a 3D mesh based on MRI voxels. Finally, Piatti et al.^13^ developed an innovative method to compute WSS based on 2D and 3D Sobel filters.

PC MRI-derived WSS calculation methods are often validated with *Computational Fluid Dynamics* (CFD) techniques in realistic vascular geometries. Nevertheless, CFD employment is limited in complex physiological conditions^14^. Alternatively, in *Laser Doppler Velocimetry* (LDV) campaigns, it is possible to reproduce the same experimental conditions of in vitro PC MRI acquisitions in terms of geometry, flow rate, pressure, fluid viscosity, and density, providing accurate velocity profiles to serve as a reference for PC MRI data. LDV enables an accurate measurement of absolute velocity components on a single point with the drawback of having to move the measurement volume to obtain velocity mapping, and it represents an intrinsic, robust velocimetry technique since velocity data are obtained through robust statistical post-processing tools. In the scientific literature, few works about LDV validation for PC MRI-derived WSS computation are reported. Bauer et al.^15^ successfully investigated WSS accuracy from PC MRI data with LDV in a simplified model of an aortic aneurysm, employing in parallel CFD for direct comparison.

The present work aims to develop a robust method based on a hydraulic circuit, in which LDV served as a standard for velocity measurements, to be employed as a reference to assess the accuracy of 2D PC and 4D Flow MRI velocity acquisitions. This method is intended to be employed to evaluate any potential developments in acquisition and flow quantification protocols, particularly for the calculation of WSS. Firstly, a hydraulic test rig was designed and manufactured to be MRI-compatible and to allow accurate LDV measurements under both steady and pulsatile flow conditions. Next, velocity data obtained with the test rig were statistically analyzed to assess the accuracy and reproducibility of results between the two velocimetry techniques. The correspondence between LDV and PC MRI WSS profiles was statistically assessed. Finally, the efficacy of the method developed in this work was tested evaluating the influence of 4D Flow MRI spatial resolution on WSS profiles.

## Methods

### Experimental set-up

**Figure 1:**
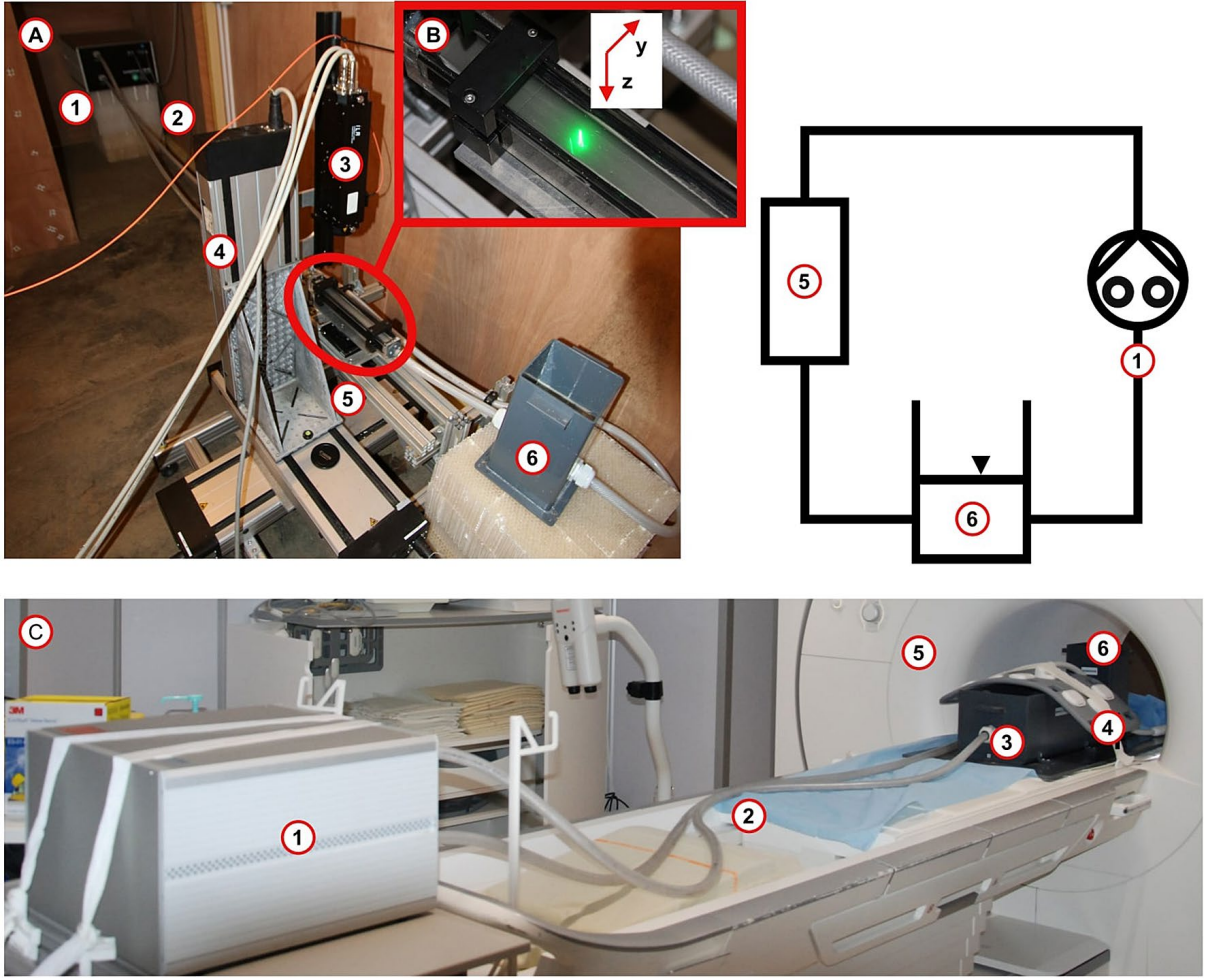
(**a**) Experimental set-up in LDV facility and its schematic (top, right): (1) Pulsatile Pump, (2) Connecting pipes, (3) LDV head, (4) Laser displacement system, (5) Test Section, (6) Reservoir. (**b**) LDV acquisitions. (**c**) Experimental Set-up in MRI facility: (1) Pulsatile Pump, (2) Connecting pipes, (3) Agarose gel plastic box with the test section, (4) MRI array coils, (5) MRI system, (6) Reservoir.

The experimental set-up (Fig. 1) was designed to keep limited dimensions, to be easy to move between facilities, and to be compatible with LDV and MRI constraints. It was based on an MRI-compatible gear pump (CardioFlow 5000, Shelley Medical Technology), a test section and a reservoir open to the atmospheric pressure (Fig. 1a), completed with several connecting flexible PVC pipes for ease of assembly and transport between LDV and MRI facilities. The pump provided a TTL signal to synchronize both LDV and MRI acquisitions, the period of flow rate signals was $T_c$ = 1 s for both steady and pulse acquisitions. Made of a mixture of 40% by weight of glycerol in water, the working fluid reproduced blood density ($\rho$ = 1098 ${\mathrm {kg/m}}^3$) and viscosity ($\mu$ = 0.00345 ${\mathrm {Pa{\cdot}s}}$) closely^16^. The test section was a compact $25 \times 25 \times 345$ mm squared PMMA pipe (Fig. 1b). The measurement area was located on a cross-section placed 265 mm downstream of the test section inlet, to limit the influence of the test section inlet and outlet. Investigations were performed under laminar steady and pulse flow conditions, deploying an ultrasound flowmeter (Flowmax 44i, MIB Gmbh) to ensure the flow rate control in the measurement location, $Q_{\mathrm {exp}}$ ($Q_{\mathrm {exp}} = 98.67$ ml/s and $Q_{\mathrm {exp}} = 16.47 + 35.64\sin {(2\pi t + 0.50)}$ ml/s for steady and pulse flow rate respectively). During MRI measurements, a plastic box filled with agarose gel (A8963, Applichem) surrounded the test section to reproduce human body tissues and to reduce noises (Fig. 1c).

### Phase contrast MRI

Firstly, one standard 2D PC and one standard 4D Flow MRI sequence were used on a 1.5 T MRI system (Aera 1.5T, Siemens), their parameters were selected as in current clinical practice, defining then tests called $Steady2D_{X0}$, $Pulse2D_{X0}$, $Steady4D_{X0}$, and $Pulse4D_{X0}$ (Table 1)^17^. In view to fit the maximum expected velocity $v_{max}$, the *Velocity ENCoding* (*VENC*) was adjusted as in Eq. (1) ($v_{max}=28$ cm/s and $v_{max}=12$ cm/s for steady and pulse tests respectively).

1 $$\begin{aligned} \textit{VENC}\approx {\left\{ \begin{array}{ll} v_{max} +10\%v_{max}\hspace{3pt} \text {for steady tests}\\ v_{max} +30\%v_{max}\hspace{3pt} \text {for pulse tests} \end{array}\right. } \end{aligned}$$

Next, a second experimental campaign was performed employing the 4D Flow sequence. The size of the voxels was progressively reduced from $2.22 \times 2.22 \times 2$ to $1.86 \times 1.86 \times 1.86$, and then to $1.50 \times 1.50 \times 1.50$ mm. The refinement was carried out with isometric voxels to simplify the analysis; the starting size was considered as “almost” isometric since the smallest dimension was shorter than the others by about 10%. Consequently, the Field of View (FOV) was proportionally scaled. Acquisition parameters are resumed in Table 1, defining instead tests subsequently called $Steady4D_{X1}$, $Steady4D_{X2}$, $Pulse4D_{X1}$, and $Pulse4D_{X2}$. Markers on the experimental set-up enabled the placement of the measurement location at the center of the MRI scanner, drastically reducing the effects of the magnetic field inhomogeneities. The velocity signal from the static homogeneous region composed by the agarose gel was exploited to define the impact of the velocity offset from magnetic field inhomogeneities and noise. The distribution of this signal was a Gaussian with a non-zero mean and variance, representing the phase offset and the velocity noise respectively. As a first approximation, the influence of the offset was considered negligible; its ratio with the mean of the velocity signal (${\overline{v}}_{\mathrm {fluid}}$) in the fluid domain was lower than 3%. To evaluate the impact of noise on PC MRI acquisitions, *Velocity to Noise Ratio* (*VNR*) was defined as in Eq. (2) (${\sigma _{{\text{ v }}_{\text{ gel }}}}$ is the variance of velocity noise in the agarose gel calculated as the standard deviation of the velocity signal of pixels in the agarose gel).

2 $$\begin{aligned} \textit{VNR}=\frac{{\overline{v}}_{\mathrm {fluid}}}{{\sigma _{\mathrm {v}}}_{\mathrm {gel}}} \end{aligned}$$

**Table 1:** Left: Standard 2D and 4D PC MRI experimental protocols. Right: Spatial resolution refinement 4D PC MRI protocol. X0 original data resolution, X1 data resolution refinement 1, X2 data resolution refinement 2.

Test $\rightarrow$	Experimental campaign 1				Experimental campaign 2: spatial resolution refinement			
	2D PC MRI		4D flow MRI		4D flow MRI			
	${Steady2D_{X0}}$	${Pulse2D_{X0}}$	${Steady4D_{X0}}$	${Pulse4D_{X0}}$	${Steady4D_{X1}}$	${Pulse4D_{X1}}$	${Steady4D_{X2}}$	${Pulse4D_{X2}}$
Resolution (mm)	$1.17 \times 1.17$		$2.22 \times 2.22$		$1.86 \times 1.86$		$1.5 \times 1.5$	
Slice	1		32		32			
Thickness (mm)	6		2		1.86		1.5	
Flip angle ($^\circ$)	20		7		7			
Excitations	1				1			
Averages	1				1			
Gating	R/P				R/P			
Phases	30				30			
FOV (mm)	$159 \times 300$		$288 \times 319$		$238 \times 268$		$192 \times 216$	
Matrix (px)	$136 \times 256$		$32 \times 130 \times 144$		$32 \times 128 \times 144$		$32 \times 128 \times 144$	
VENC (cm/s)	30	16	30	16	30	16	30	16
TE (ms)	3.73	4.72	3.49	4.28	3.53	4.31	3.57	4.36
TR (ms)	48.16	56.08	48.32	54.64	48.72	54.96	49.04	55.36
TA (min:s)	0:44		6:48		6:53		7.11	
Temp Res (ms)	200		250		250			

Resolution refers to *z*–*y* axes and matrix refers to the number of pixels along *z*, *y*, and *x* axis respectively; see Fig. 1b for axis reference.

*R/P* retro/pulse, *TE* echo time, *TR* repetition time, *TA* acquisition time, *Temp Res*, temporal resolution.

### Laser doppler velocimetry

For LDV investigations, a low concentration ($C_{\mathrm {volume}} \approx$ 0.05 %) of 10 $\upmu$m silver-coated hollow glass spheres (S-HGS, Dantec) was added to the mixture. They exhibited a high refractive index, increasing the LDV SNR with respect to non-coated hollow glass sphere seeding. The relative density gap with the fluid ($\rho _{\mathrm{fluid}}/\rho _{\mathrm{particle}} \approx 0.70$) did not induce undesirable behaviors like a lift or gravitational effects, particle accumulation, or modifications of the working fluid physical properties. The axial velocity along the *x*-axis (see Fig. 1b for axes reference) was measured by an LDV system (fp50 LDV system). The optical path of the laser beams passing through the upper face of the test section and the fluid was defined according to Snell’s refraction law (Fig. 1b) (Eq. 3, $\alpha$ represents the incidence, in the air, or refraction, in the fluid, angle, and *n* the refractive index).

3 $$\begin{aligned} n_{\mathrm {air}}\sin {\alpha _{\mathrm {air}}}=n_{\mathrm {fluid}}\sin {\alpha _{\mathrm {fluid}}} \end{aligned}$$

The location of a measurement point was calculated considering the refractive index mismatch at the optical interface along the *z*-direction, discounting the window thickness. To optimize the whole acquisition time, measurements were obtained on a square grid of $63 \times 63$ points equally spaced by 0.40 mm; this length was the LDV data spatial resolution. For each measurement point, the velocity was recorded for 30 s and velocity data were obtained with a home-made Matlab script (Matlab R2016b, MathWorks). For steady tests, a *two-class Gaussian Mixture Model* was applied to the velocity sample distribution to separate the signal Gaussian distribution, determining the mean velocity and measurement uncertainty $\sigma _{\mathrm{s}}$, from velocity outliers, determined during zero flow rate measurements. For pulse acquisitions, the velocity signal was sampled in periods as long as those of the pump flow rate; all periods were overlapped and finally fitted with a sinusoid, according to the Womersley theory of velocity profiles under pulse flow conditions, for which velocity at each point has the same waveform of the inlet flow rate^18^. The coefficient of determination $R^2$ (best fit for $R^2$ = 1) was employed to assess the uncertainty of the measurement. A CFD verification study was performed to preliminary control LDV measured velocity profiles on a simple test case for which CFD is deemed reliable. Numerical simulations were performed using the Finite Volume Method (Star CCM+, Siemens). Calculations were based on a Cartesian mesh of 1725000 0.5mm-size cubic elements and results were then linearly interpolated to fit the LDV grid (Matlab R2016b, MathWorks) for direct comparison. For the Steady test, the mean value of $\sigma _{\mathrm{s}}$ was $6.63 \times 10^{-4}$ m/s, while for the Pulse test the mean value of $R^2$ was 0.82. The *Locally Weighted Scatterplot Smoothing* (LOWESS) method (Matlab R2016b, MathWorks) was applied to experimental data to find velocity profile boundaries, smooth noises, and scale results in a uniform grid. Ultimately, a broader LDV grid was generated to approach a similar resolution of MRI data to allow direct comparison, with the average of all LDV points contained in a window with the identical size of one MRI pixel.

### Wall shear stress calculation

*WSS* is defined as (4)

4 $$\begin{aligned} WSS = \mu \left. \frac{\partial \vec {v}}{\partial n}\right| _{wall} \end{aligned}$$

where $\mu$ is the blood dynamic viscosity, $\vec {v}$ the blood velocity, and $\vec {n}$ the normal direction to the arterial wall. The methods proposed by Stalder et al.^10^ (2D-WSS) and Potters et al.^11^ (3D-WSS) were employed to assess WSS from PC MRI and LDV data-sets in the same location of the test section, in view to demonstrate the efficacy of this experimental system to validate PC MRI post-processing algorithms for WSS computation. For 2D-WSS, from 2D PC or sliced 4D Flow MRI data, the lumen is contoured through B-splines and within this fluid domain, the blood velocity profile is interpolated with B-splines from which its derivative is calculated. In the 3D-WSS method instead, velocity derivatives are calculated on the 3D lumen segmentation from B-splines interpolation of blood velocity profiles, exploiting only a fixed number of velocity points close to the boundary.

Both methods were tested independently of the lumen segmentation technique. The lumen segmentation was obtained from the test section CAD (SolidWorks 2016, Dassault Systèmes) as an *stl* file overlapping the test section fluid domain in the anatomy images. Markers on the TS permitted to obtain data from different datasets at the same location. The obtained data were employed as inputs for 2D-WSS. 3D-WSS calculations need to be performed from 3D data sets. For 4D acquisitions, such 3D data sets are readily available, but it is not the case for the two dimensional LDV and 2D PC MRI measurements. Thus, for these two latter, 3D data sets were artificially built by duplicating the 2D measurements in several planes in the *x*-axis (see Fig. 1b for axes reference), spaced by 2 mm as in the 4D data sets. For 4D Flow MRI data resolution refinement, WSS was calculated only with the 3D-WSS method.

For the comparison between LDV and CFD, WSS was calculated with a first-order finite difference scheme (Eq. 5, $\mu$ is the fluid viscosity, $\Delta s$ the spatial resolution, $v_{wall}$ and $v_{wall+1}$ the velocity at the boundary and at the boundary nearest point, respectively).

5 $$\begin{aligned} WSS_{FD} = \mu \frac{v_{wall+1} - v_{wall}}{\Delta s} \end{aligned}$$

### Statistical analysis

Flow Rate (*Q*) and WSS contour mean (${\overline{WSS}}$) were firstly computed through the integration of velocity and WSS profiles. For PC MRI data, *Q* and ${\overline{WSS}}$ were calculated for each phase and then for Steady test they were averaged. Flow rate errors were calculated against values provided by the flowmeter $Q_{\mathrm {exp}}$, while for ${\overline{WSS}}$ they were calculated with LDV as reference (Eq. 6, s and p refer to Steady and Pulse test respectively).

6 $$\begin{aligned} \epsilon _Q^{\mathrm {s}} &= 100\frac{\mid Q_{\mathrm {MRI/LDV}} - Q_{\mathrm {exp}} \mid }{Q_{\mathrm {exp}}} \% \\ \epsilon _{WSS}^{\mathrm {s}} &= 100\frac{\mid {\overline{WSS}}_{\mathrm {MRI}} - {\overline{WSS}}_{\mathrm {LDV}} \mid }{{\overline{WSS}}_{\mathrm {LDV}}} \% \\ \epsilon _Q^{\mathrm {p}} &=100\sqrt{\frac{\sum _{i=1}^{30}(Q^{\mathrm {MRI/LDV}}_i-Q^{\mathrm {exp}}_i)^2}{\sum _{i=1}^{30}(Q^{\mathrm {exp}}_i)^2}}\% \\ \epsilon _{WSS}^{\mathrm {p}}&=100\sqrt{\frac{\sum _{i=1}^{30}({\overline{WSS}}^{\mathrm {MRI}}_i-{\overline{WSS}}^{\mathrm {LDV}}_i)^2}{\sum _{i=1}^{30}({\overline{WSS}}^{\mathrm {LDV}}_i)^2}}\% \end{aligned}$$

Linear regression $Y=aX+b$ was performed for velocity and WSS profiles, $X=[x_i,\ldots ,x_N]$ and $Y=[y_i,\ldots ,y_N]$ were respectively the *N* velocity points of LDV and PC MRI datasets. Coefficients *a* and *b* were determined for the analysis. Additionally, Root Mean Square Error (*RMSE*), Coefficient of Determination $R^2$, and L2 norm error $\epsilon _{\mathrm {L2}}$ were included in the analysis (Eq. 7)

7 $$\begin{aligned} RMSE = \sqrt{\frac{1}{N}\sum _{i=1}^{N}\Big (\frac{y_i - x_i}{x_i}\Big )^2} \nonumber \\ R^2 = 1 - \frac{\sum _{i=1}^{N}(y_i - x_i)^2}{\sum _{i=1}^{N}(y_i - {\overline{y}})^2} \nonumber \\ \epsilon _{\mathrm {L2}}=100\sqrt{\frac{\sum _{i=1}^{N}(y_i-x_i)^2}{\sum _{i=1}^{N}(x_i)^2}}\% \end{aligned}$$

Correlation between different velocity or WSS data was assessed with the Pearson Correlation Coefficient (*PCC*), while consistency was assessed with Intraclass Correlation Coefficient (*ICC*). Coefficients *a* and *b*, *RMSE*, $R^2$, $\epsilon _{\mathrm {L2}}$, *PCC*, and *ICC* were evaluated for each acquired phase and subsequently averaged over the 30 samples. Their scores for the exact correspondence between diverse groups of data are reported in Table 2.

**Table 2:** Statistical analysis: perfect correspondence values for correlation (by *PCC*) and consistency (by *ICC*) scores.

Correlation			Consistency^19^		
PCC $\le$ 0.3	None	−−	ICC $\le$ 0.5	Poor	−−
0.3 < PCC $\le$ 0.5	Weak	−	0.5 < ICC $\le$ 0.75	Moderate	−
0.5 < PCC $\le$ 0.7	Moderate	+	0.75 < ICC $\le$ 0.9	Good	+
PCC > 0.7	Strong	++	ICC > 0.9	Excellent	++

## Results

### Preliminary verification of LDV with CFD

**Figure 2:**
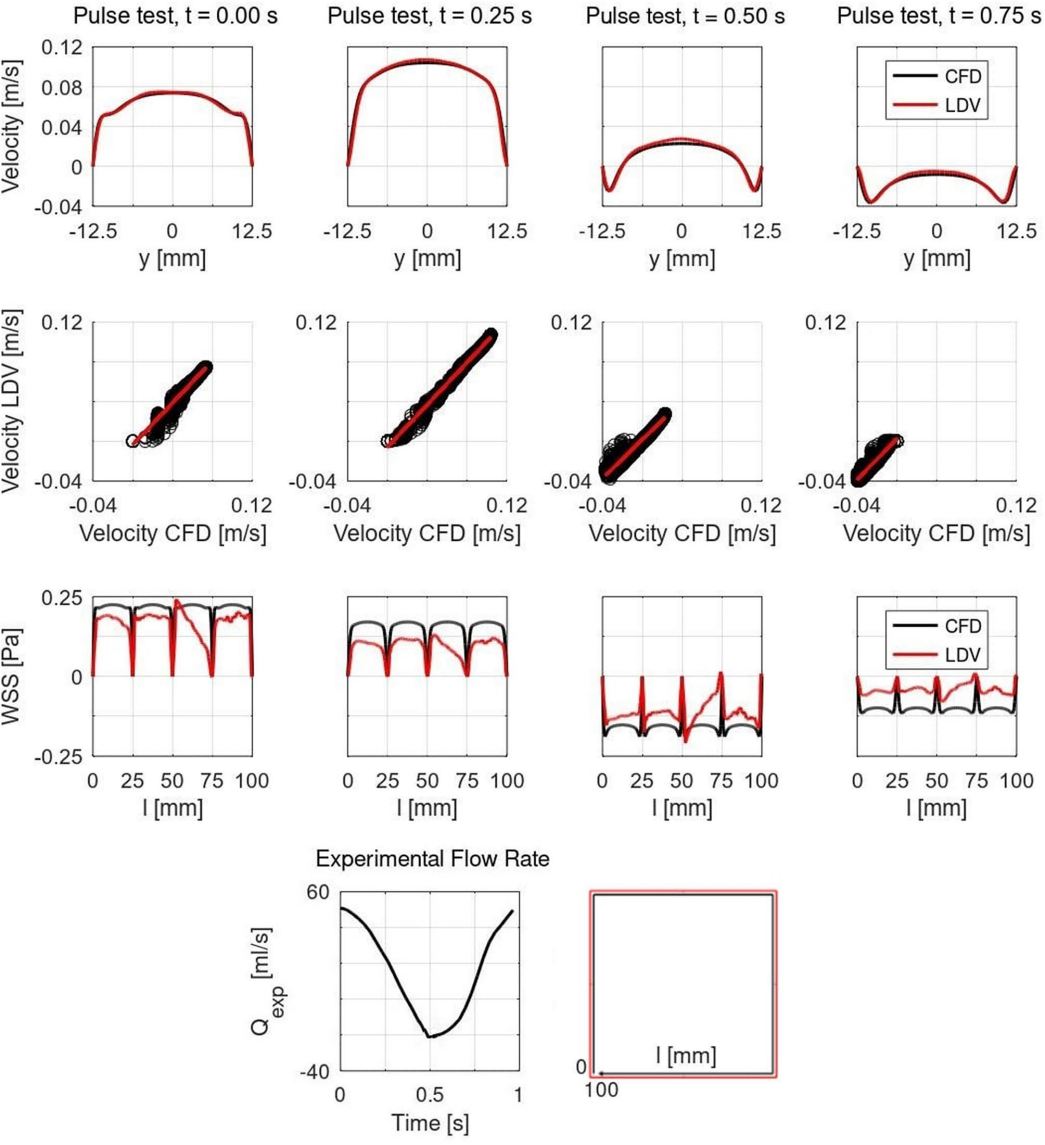
LDV verification with CFD for pulse test at different time frames. First row: Velocity centerlines; Second row: Correlation plots with regression lines in red; Third row: Finite difference WSS profiles on the test section boundaries expressed in Pa, results are plotted along the boundary coordinate *l*, $0\le l\le 100$ mm; Fourth row: Flow rate (left) and schematic of the boundary coordinate *l* to plot WSS profiles (right).

Results of the LDV verification with CFD are displayed for the pulse test in Fig. 2. Velocity profiles show a good agreement between LDV and CFD, as well as correlation plots with regression lines. WSS profiles display significant discrepancies between CFD and LDV, as CFD did not reproduce the exact same experimental conditions. Specifically, results for values of the boundary coordinate *l* between 25 and 75 mm might suggest that an imperfect alignment of the flow at the experimental test section inlet occurred.

### PC MRI velocity profiles

**Figure 3:**
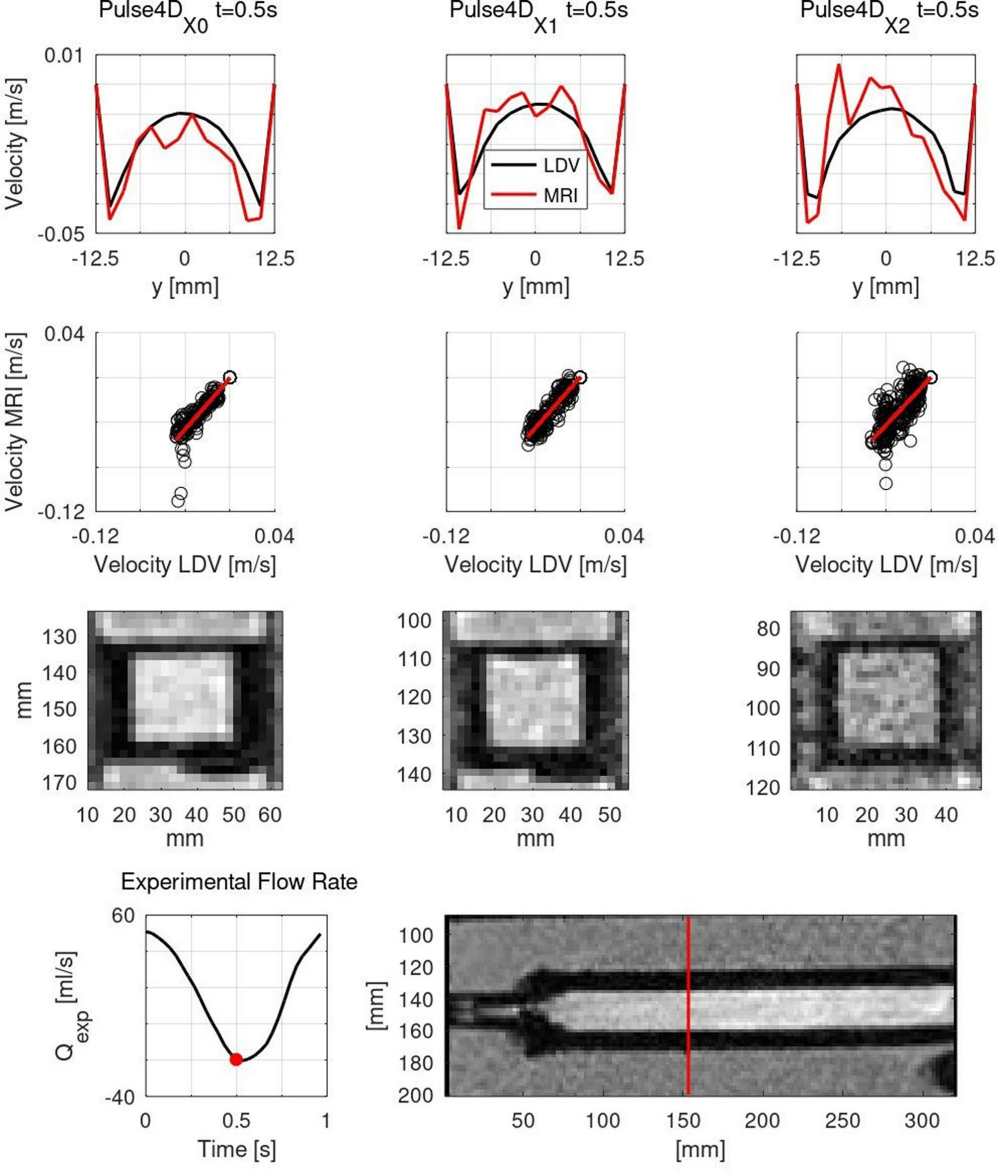
PC MRI Velocity Profiles, columns from left to right: ${\mathrm {Pulse4D}}_{\mathrm{X0}}$, ${\mathrm {Pulse4D}}_{\mathrm{X1}}$, ${\mathrm {Pulse4D}}_{\mathrm{X2}}$ tests. First row: Velocity centerlines; Second row: Correlation plots with regression lines in red; Third row: Magnitude images; Fourth row: Flow rate and location of measurement cross-section in the test section, marked with the red line.

Results for velocity data centerlines and correlation plots between LDV and PC MRI are reported in Fig. 3 for ${\mathrm {{Pulse4D}}}_{\mathrm{X0}}$, ${\mathrm {Pulse4D}}_{\mathrm{X1}}$, and ${\mathrm {Pulse4D}}_{\mathrm{X2}}$ tests (see Table 1 for test labels), while the quantitative analysis is summarized in Table 3. The effect of the resolution refinement on the magnitude images is displayed in Fig. 2. Even though the refinement improved the image resolution, for the fine resolution, overall image quality decreased. There was globally a good agreement between LDV and PC MRI results. Both 2D PC and 4D Flow MRI reproduced LDV velocity profiles well, and the same feature was found with the refinement of the spatial resolution in 4D acquisitions. 2D PC MRI results were noisier than those of 4D Flow MRI. Noise impacted more data close to the boundaries and at low flow rate phases, as evidenced by the velocity oscillations. Concerning the quantitative analysis, the *VNR* was higher for 4D Flow than 2D PC MRI, and for steady than pulse acquisitions. Moreover, it decreased with voxel refinement in both steady and pulse acquisitions. For pulse acquisitions, flow rate errors were lower for 2D PC than 4D Flow MRI. While refining 4D Flow data resolution, it decreased to values comparable to those of 2D PC MRI. ${\mathrm {Pulse4D}}_{\mathrm{X1}}$ test recorded the most satisfactory performance. For the other statistical parameters, 2D PC and 4D Flow MRI results registered similar scores. Specifically, the correlation and consistency between LDV and MRI data were strong and excellent for steady tests and strong and good for pulse tests, for both MRI techniques. Results related to ${\mathrm {Steady4D}}_{\mathrm{X1}}$, ${\mathrm {Steady4D}}_{\mathrm{X2}}$, ${\mathrm {Pulse4D}}_{\mathrm{X1}}$, and ${\mathrm {Pulse4D}}_{\mathrm{X2}}$ tests displayed similar figures to those of ${\mathrm {Steady4D}}_{\mathrm{X0}}$ and ${\mathrm {Pulse4D}}_{\mathrm{X0}}$ tests. Despite these small figures, data resolution refinement improved the correspondence between LDV and MRI datasets. For these results, the correlation and consistency between LDV and MRI data were strong and excellent respectively.

**Table 3:** Statistical analysis for velocity results. Left: Standard PC MRI protocol. Right: 4D Flow MRI data resolution refinement protocol. X1 resolution refinement 1, X2 resolution refinement 2.

Test $\rightarrow$	2D PC MRI		4D Flow MRI		4D Flow MRI			
	${Steady2D_{X0}}$	${Pulse2D_{X0}}$	${Steady4D_{X0}}$	${Pulse4D_{X0}}$	${Steady4D_{X1}}$	${Steady4D_{X2}}$	${Pulse4D_{X1}}$	${Pulse4D_{X2}}$
${\epsilon _Q}$ [%]	0.93	9.34	8.96	16.33	0.50	0.51	10.61	14.09
VNR	2.27	1.28	16.39	8.88	8.84	5.99	3.86	3.10
a	1.02	1.05	0.95	0.93	0.99	1.01	0.99	1.00
b	-0.008	-0.001	-0.008	-0.003	-0.004	-0.008	-0.001	-0.003
RMSE	0.029	0.018	0.028	0.012	0.022	0.027	0.006	0.011
$R^2$	0.87	0.57	0.89	0.69	0.93	0.90	0.88	0.75
$\epsilon _{\mathrm{L2}}$	17.94	64.03	20.03	56.38	14.31	16.81	28.03	43.51
PCC	0.93 (++)	0.74 (++)	0.94 (++)	0.81 (++)	0.97 (++)	0.95 (++)	0.94 (++)	0.86 (++)
ICC	0.96 (++)	0.8 (+)	0.97 (++)	0.86 (+)	0.98 (++)	0.97 (++)	0.96 (++)	0.91 (++)

### PC MRI wall shear stress

**Figure 4:**
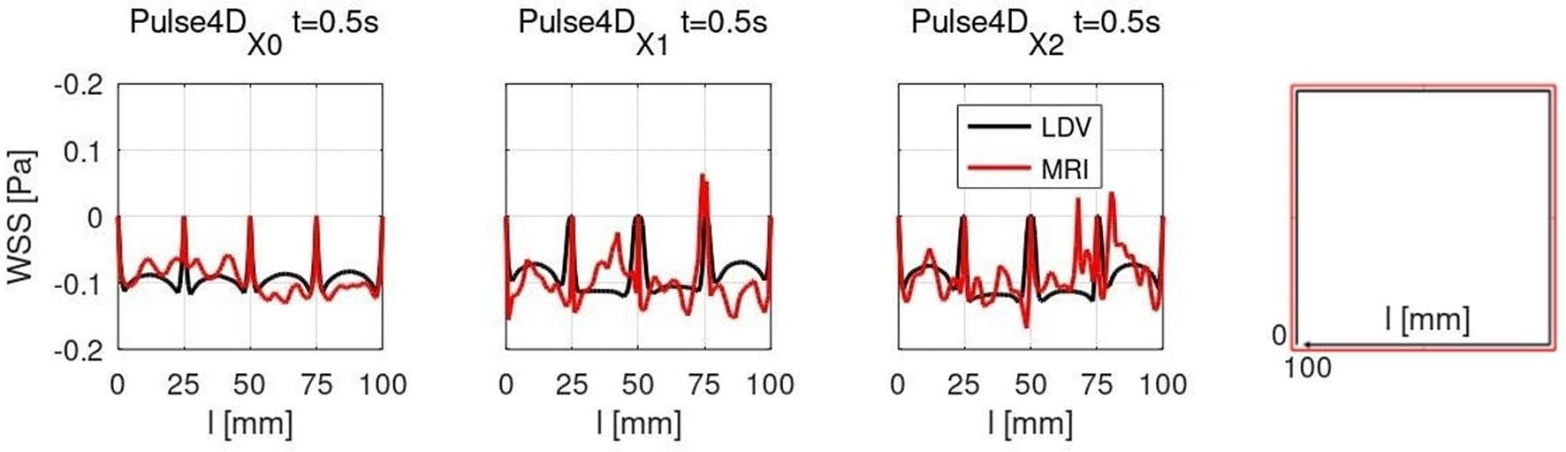
3D-WSS profiles on the test section boundaries expressed in Pa, results are plotted along the boundary coordinate *l*, $0\le l\le 100$ mm. Right: Schematic of the boundary coordinate *l* to plot WSS profiles.

Results of the WSS assessment are reported in Fig. 4 and in Table 4. Profiles of 3D-WSS calculated from 4D Flow MRI data displayed generally a satisfactory agreement with those obtained from LDV velocity data. The refinement of the voxel size introduced oscillations in 3D-WSS profiles, particularly for ${t \ge 0.5 T_c}$. Concerning WSS quantitative analysis, overall results for 2D-WSS were inconsistent, particularly for 4D Flow acquisitions, for which figures of mean WSS errors increased importantly. Also, for Steady tests, correlation and consistency were weak and moderate respectively, while for Pulse tests, there was no correlation and consistency was poor. Results of 3D-WSS instead recorded better performances. Figures were in the same order of magnitude for 2D PC and 4D Flow MRI, but the results of pulse acquisitions were slightly worse. For Steady tests, correlation was strong and consistency excellent while, for Pulse tests, consistency was moderate and consistency was good for 4D Flow and moderate for 2D PC MRI. For data resolution refinement, results registered improved performances for steady tests, and scores worsened with the decrease of the voxel size. However, for steady tests, consistency and correlation remained excellent and strong respectively.

**Table 4:** Statistical analysis for 2D-WSS and 3D-WSS results.

	2D-WSS				3D-WSS			
	${Steady2D}_{{X0}}$	${Pulse2D}_{{X0}}$	${Steady4D}_{{X0}}$	${Pulse4D}_{{X0}}$	${Steady2D}_{{X0}}$	${Pulse2D}_{{X0}}$	${Steady4D}_{{X0}}$	${Pulse4D}_{{X0}}$
${\epsilon _{WSS}}$ [%]	22.76	19.84	70.67	109.70	2.62	7.33	0.83	15.90
a	0.70	0.84	0.40	0.06	0.99	0.96	0.91	0.79
b	0.022	-0.003	-0.001	0.006	0.010	0	0.027	0.002
RMSE	0.084	0.047	0.012	0.036	0.041	0.037	0.026	0.023
$R^2$	0.39	0.20	0.016	0.030	0.76	0.37	0.88	0.48
$\epsilon _{\mathrm{L2}}$	34.35	82.72	74.19	201.95	13.85	50.77	9.00	41.58
PCC	0.63 ${(+)}$	0.42(-)	0.39(-)	0.035 (–)	0.87 ${(++)}$	0.58 ${(+)}$	0.94 ${(++)}$	0.65 ${(++)}$
ICC	0.77 ${(+)}$	0.47 (–)	0.55(-)	0.014 (–)	0.93 ${(++)}$	0.64(-)	0.97 ${(++)}$	0.74(-)

	3D-WSS							
	${Steady4D}_{{X1}}$	${Steady4D}_{{X2}}$	${Pulse4D}_{{X1}}$	${Pulse4D}_{{X2}}$				
${\epsilon _{WSS}}$ [%]	5.80	3.64	13.45	28.10				
a	0.82	0.98	0.70	0.52				
b	0.061	0.011	0.013	0.007				
RMSE	0.049	0.086	0.030	0.042				
$R^2$	0.75	0.58	0.44	0.24				
$\epsilon _{\mathrm{L2}}$	20.01	31.16	47.87	70.58				
PCC	0.87 (++)	0.76 (++)	0.63 (+)	0.40 (−)				
ICC	0.93 (++)	0.84 (+)	0.74 (−)	0.49 (−−)				

Top: Standard PC MRI protocol. Bottom: 4D Flow MRI data resolution refinement protocol. X1 resolution refinement 1, X2 resolution refinement 2.

## Discussions

In this work, an easy-to-use and accurate LDV-based system to validate potential improvements in PC MRI acquisitions and post-processing was designed and manufactured. The reliability of the system was preliminarily assured through the successful assessment of LDV measurement accuracy and errors. The experimental campaign was carried out proficiently, proving the efficacy of the employment of this sort of tool in research on clinical applications of PC MRI. Despite it being time-consuming, LDV enabled accurate measurements of fluid velocity profiles, providing information to test preliminary PC MRI performances and thus flow quantification, as it did for WSS calculation. Even though LDV and PC MRI campaigns were performed independently at different times, on the same test rig but in separated facilities, a good level of reproducibility of the results with the two different techniques was achieved for both velocity and WSS results. Accurate LDV tools represent, therefore, a robust alternative to CFD to simply assess 4D Flow MRI quality. Although CFD is often employed as a reference for PC MRI, its role of ground truth is not widely accepted in the scientific community.

For velocity profiles, results generally showed a strong correlation and an excellent consistency between PC MRI and LDV, representing a strong basement for further investigations on post-processing algorithms, as evidenced by statistical parameter scores. Firstly, a method was employed to degrade the LDV data resolution to directly compare LDV and MRI velocity profiles, inducing a small distance shift $d_{\mathrm {shift}}$ between LDV and MRI grids, but without a noticeable effect on the analysis (${d_{\mathrm {shift}}}_{\mathrm {max}}$ = 0.2632 mm). No significant differences were noticed between standard 2D and 4D acquisitions, except for flow rate errors that were greater for 4D Flow MRI results since data resolution was lower. As largely discussed, validation of WSS calculation techniques with this experimental set-up represents a crucial clinical aspect. To demonstrate that, the system was proved with two algorithms to compute WSS from PC MRI data. WSS calculation from PC-MRI-based velocity data in this specific application was positively accomplished. Even if in clinical practice PC MRI data processing includes segmentation of anatomical images to obtain the domain in which blood flow quantification is performed, the analysis was simplified by testing the methods independently of the segmentation technique. The improved accuracy of 3D-WSS and its greater performances and versatility compared to 2D-WSS have been properly assessed thanks to this LDV based tool. For 3D-WSS, there were no significant differences between 2D PC and 4D Flow MRI results, while 4D Flow 2D-WSS outcomes were more inaccurate than 2D PC 2D-WSS. This high sensitivity of the 2D-WSS method to the velocity data resolution might be due to the employment of a squared geometry, representing a sensitive case for B-spline interpolation for the corners of lumen contour.

Finally, a concrete application of this approach to solve a relevant clinical challenge in 4D Flow MRI acquisitions and derived WSS calculation was investigated to additionally prove the benefit of relying on LDV reference data to optimize 4D Flow MRI. The influence of voxel size on acquired velocity data and computed 3D-WSS was successfully assessed. For velocity profiles, the agreement between LDV and 4D Flow in terms of flow rate errors, consistency, and correlation globally improved with the data resolution refinement. For the finest data resolution compared to the medium one, this agreement worsened though, which might be attributed to a higher velocity noise consequently to the voxel size reduction. This would also explain why the agreement between LDV and 4D flow MRI worsens when refining the resolution for 3D-WSS profiles, which are more sensitive to noise than velocity profiles. The impact of noise is stronger for low-velocity data, like those close to the boundaries with which WSS is calculated. This might have resulted in a reduction of the accuracy of the WSS assessment caused by a significant increase in noise while refining the spatial resolution.

It can be revealed from this experimental campaign that, as a general rule in clinical practice, for an equivalent acquisition time there is a trade-off to be found between data spatial resolution and *VNR*, as noise has a stronger impact on WSS calculation with respect to the velocity measurement. For potential clinical applications of current WSS computation techniques, an MRI spatial resolution improved with respect to that of standard protocols might not always imply a more accurate assessment of WSS, if *VNR* is highly degraded by an increase of noise in the acquired data. In this study, the effects of the temporal resolution were not investigated, but the system could additionally be employed to accomplish this task.

Limitations of the present study included the employment of an unphysiological scenario, in terms of test section shape and flow rate conditions, and the background phase removal. The selection of a squared test section did not represent a realistic anatomical geometry. Nevertheless, it represented a simple and optimal set up for accurate LDV measurements, since the optical interface crossed by LDV laser beams is constituted of a flat-screen, preventing then optical aberrations, which could instead arise from a curved surface. Therefore this squared test section was well suited to investigate the WSS biomarker. The flow rate curves were selected to reproduce the thoracic aorta fluid dynamic conditions in terms of Reynolds ($Re_{\mathrm {mean}} \approx 1100$) and Womersley (${\alpha \approx 20}$) numbers, compatibly with the experimental set-up constraints ($Re_{\mathrm {mean}} = 1273$ for Steady tests; $Re_{\mathrm {mean}} =190$ and $\alpha =18$ for Pulse tests). These unphysiological flow rate conditions led to lower magnitudes of WSS signals, which were thus harder to assess. Nonetheless, the proposed methodology is valid for the higher WSS magnitude found in physiological flow rate conditions. Further experimental campaigns with a more realistic scenario of flow rate and vessel geometry could be performed, however, with this experimental device. Concerning the unphysiological flow rate conditions, for steady tests, the approximate average fluid dynamic conditions of the thoracic aorta were achieved. For pulse tests instead, through the Womersley number, only the pulsatile features of blood flow of this specific cardiovascular location were replicated. In any case, even if during pulse tests velocity magnitudes were far from physiological values, this represented a challenging scenario for the assessment of WSS, since velocity gradients were lower than physiological values. Further experimental campaigns with a more realistic scenario of flow rate and vessel geometry will be performed.

Lastly, preliminary PC MRI processing procedures to remove the effects of magnetic field inhomogeneities and other artefacts are mandatory in clinical investigations, but in this specific application, they were not implemented. Their effects were evaluated considering velocity data in the agarose gel surrounding the test section, and they were considered negligible in a first approximation. This might be explained by the fact that in this straightforward application the region of interest was located in the center of the scanner where magnetic fields are more homogeneous, and the FOV was properly centered in this region. Notwithstanding no correction was applied, results showed a good agreement with other experimental references (LDV, flowmeter), and WSS assessment was properly conducted. In this first preliminary phase, this aspect was not considered crucial, but it will be considered for any other further investigations involving this experimental set-up.

This work represents a strong foundation for future validation of novel developments for blood flow quantification from PC MRI acquisitions. Particularly, the method based on laser Doppler velocimetry presented here might provide reliable information about the accuracy, reproducibility, and applicability of the techniques employed to calculate in vivo WSS from 4D Flow MRI data. Based on the findings of this work, future developments of the present study are represented by the design of a novel hydraulic rig under more realistic conditions in terms of physiological and pathological anatomies, flow rates, and pressure. It could be employed as an independent standard to validate 4D Flow MRI acquisition sequences and post-processing, particularly regarding quantitative hemodynamic biomarkers for blood flow quantification, such as the wall shear stress, pulse wave velocity, or pressure mapping.

## Data Availability

The datasets generated during and/or analysed during the current study are available in the following repository https://drive.google.com/drive/folders/1mGXXfrQALE-QcAeyT63_z3LJ7KJzkc5n?usp=sharing.
