# Publisher Correction: An LDV based method to quantify the error of PC-MRI derived Wall Shear Stress measurement

**DOI:** 10.1038/s41598-021-91966-x

**Published:** 2021-06-15

**Authors:** Marco Castagna, Sébastien Levilly, Perrine Paul-Gilloteaux, Saïd Moussaoui, Jean-Marc Rousset, Félicien Bonnefoy, Jérôme Idier, Jean-Michel Serfaty, David Le Touzé

**Affiliations:** 1 LHEEA Lab, École Centrale Nantes, CNRS UMR 6598, 1 rue de la Noë, 44321 Nantes, France; 2 Université de Nantes, CHU Nantes, CNRS UMR 6291, INSERM UMR 1087, L’institut du thorax, 8 quai Moncousu, 44035 Nantes, France; 3 LS2N, École Centrale Nantes, CNRS UMR 6004, 1 rue de la Noë, 44321 Nantes, France; 4 Université de Nantes, CHU Nantes, CNRS UMS 3556, INSERM UMS 016, SFR Santé, 8 quai Moncousu, 44035 Nantes, France

Correction to: *Scientific Reports * 10.1038/s41598-021-83633-y, published online 18 February 2021

In the original version of this Article, the author David Le Touzé was incorrectly indexed. This error has now been corrected.

